# Prenatal genetic analysis and differential pregnancy outcomes of two de novo cases showing mosaic isodicentric Y chromosome

**DOI:** 10.1186/s13039-020-0472-y

**Published:** 2020-02-11

**Authors:** Si He, Hui Xi, Jing Chen, Dan Wang, Jialun Pang, Jiancheng Hu, Qin Liu, Zhengjun Jia, Hua Wang

**Affiliations:** The prenatal diagnosis center of Hunan Province, The Maternal and Child Health Hospital of Hunan Province, 53 Xiangchun Road, Kaifu District, Changsha City, Hunan Province China

**Keywords:** Isodicentric Y, AZF gene, SNP array

## Abstract

**Background:**

Fetal cells collected from the amniotic fluid of two pregnant women indicated sex chromosome abnormalities. Therefore, we performed G-banded chromosome karyotype analysis, single nucleotide polymorphism array (SNP array), fluorescence in situ hybridization (FISH), and sequence-tagged sites (STS) analysis of the Y chromosome to determine the rare molecular genetics of the two fetuses.

**Case presentation:**

The karyotypes of the fetuses from patients 1 and 2 were mos 45,X[92]/46,X,+idic(Y)(q11.21)[8] and mos 45,X[20]/46,X,+idic(Y)(q11.223)[80], respectively. Fetus 1 had a 7.76 Mb deletion in Yq11.222q11.23 and a 15.68 Mb duplication in Yp11.2q11.21. Fetus 2 had 21 Mb of repetitive segments in Yp11.3q11.223. Azoospermia factor (AZF) detection by STS analysis revealed a missing AZFb+c region in fetus 1 and three functional AZF regions in fetus 2. The isodicentric Y chromosome (idic (Y)) in both fetuses arose de novo. The pregnancy of patient 1 was terminated, whereas the fetus of patient 2 was delivered and is now 10 months old with normal appearance and growth.

**Conclusion:**

A combination of technologies such as chromosome karyotyping, FISH, SNP arrays, and STS analysis of the Y chromosome is important in prenatal diagnosis to reduce birth defect rates and improve the health of the Chinese population.

## Introduction

Sex chromosome abnormalities account for ~ 0.71% [1] of all prenatal diagnoses and can cause fetal gonadal dysgenesis, structural and functional abnormalities of other organs, and mental retardation or disorders [2]. The two fetuses in this study were both prenatally diagnosed as having sex chromosome abnormalities; both had only one X chromosome, and one additional small supernumerary marker chromosome (sSMC) with an unknown structure was found in some karyotypes. We confirmed a diagnosis using G-banded chromosome karyotyping, SNP arrays, FISH, and STS analysis of the Y chromosomes. A comparison with parental chromosomes confirmed that the form of the sSMC in both fetuses was an isodicentric Y chromosome (idic [Y]) that had arisen de novo. Furthermore, fetus 1 had no AZF b + c region and fetus 2 did not have an AZF deletion.

Idic (Y) is clinically rare, and the clinical phenotype of patients with idic (Y) is determined based on factors such as the breakpoint of the Y chromosome, the proportion of idic (Y) cell lines in the gonads and other tissues, and the presence of the SRY and AZF genes. Ultrasound showed male external genitalia [3] in both fetuses herein, but idic (Y) is associated with male gonadal dysgenesis and dyszoospermia [4, 5]. Therefore, whether the two fetuses would develop such issues postnatally was important to determine. The AZF region of the Y chromosome comprises the subregions AZFa, AZFb, and AZFc, the deletion of one or more of which, leads to dyszoospermia. After genetic counseling provided by Hunan Provincial Maternal and Child Health Care Hospital, the two pregnancies had different outcomes. One was terminated, whereas the other was carried to term. Here we describe the details of these pregnancies.

## Case presentation

### Patients

We describe two pregnant women who required prenatal diagnosis. Patient 1 was aged 22 years, and was G1P0. First- and second-trimester screens for Down syndrome revealed that her fetus was at critical risk. Each of her three noninvasive prenatal tests failed. Ultrasound during early pregnancy displayed a nuchal translucency of 1.7 mm and a crown-rump length of 48 mm, and repeated ultrasound at 24 weeks of gestation revealed that her fetus had a slightly wide posterior cranial fossa (10.4 mm), which MRI later confirmed was 15.3 mm, and the cisterna magna was not excluded.

Patient 2 was aged 32 years, G2P0, with one spontaneously aborted fetus that was not genetically analyzed. A second-trimester screen for Down syndrome, revealed high risk (1/146). Four-dimensional B-ultrasound did not detect any abnormalities.

## Methods

### Sample collection

Both patients provided written informed consent to the transabdominal collection of 40 mL (4 tubes) of amniotic fluids.

### G-banded chromosome karyotyping

G-banded chromosomes were prepared from general cultures of amniotic fluid cells. We counted 100 metaphase cells and analyzed 10 karyotypes using ZEISS MetaClient (Carl Zeiss Microscopy, Oberkochen, Germany). The karyotypes are described according to the standards of the International System for Human Cytogenomic Nomenclature (ISCN 2016). Partial cell suspensions were stored at − 20 °C.

### Single nucleotide polymorphisms arrays

We used Affymetrix CytoScan 750 K array chips incorporating 200,000 SNP markers and 500,000 copy number variation (CNV) markers that are distributed across the human genome at an average density of about 1 marker/4 kb. The arrays were run strictly according to the manufacturer’s protocol and analyzed using the Chromosome Analysis Suite. Results were interpreted with reference to public databases including the hg19 human genome, the Database of Genomic Variants (DGV), the Database of Chromosomal Imbalance and Phenotype in Humans using Ensembl Resources (DECIPHER), the Online Mendelian Inheritance in Man (OMIM), the University of California, Santa Cruz (UCSC) Genome Browser and PubMed.

### Fluorescence in situ hybridization

Fluorescence in situ hybridization proceeded using AneuVysion Multicolor DNA Probe Kits. A multicolor probe panel containing CEP 18 (white), X (green), and Y (orange) was used to detect α-satellite repeats of the centrioles of chromosomes X and Y, with normalization to the white signal of chromosome 18. Hybridization signals from chromosomes in these cells were quantified using a Leica Imaging System fluorescence microscope.

### Sequence-tagged sites of the Y chromosome

Deletions in the AZFa, AZFb, and AZFc regions of the Y chromosome were detected using Y Chromosome Microdeletions Detection Kits (Amoy Diagnostics Co., Ltd., Xiamen, Fujian, China), and the results were interpreted in strict accordance with the standards manual. Each subregion encompassed two STS; AZFa comprised Y84 and sY86, AZFb comprised sYl27 and sYl34, and AZFc comprised sY254 and sY255. The internal references were SRY and ZFX/Y.

## Results

### Cytogenetics and molecular cytogenetics

**Fig. 1:**
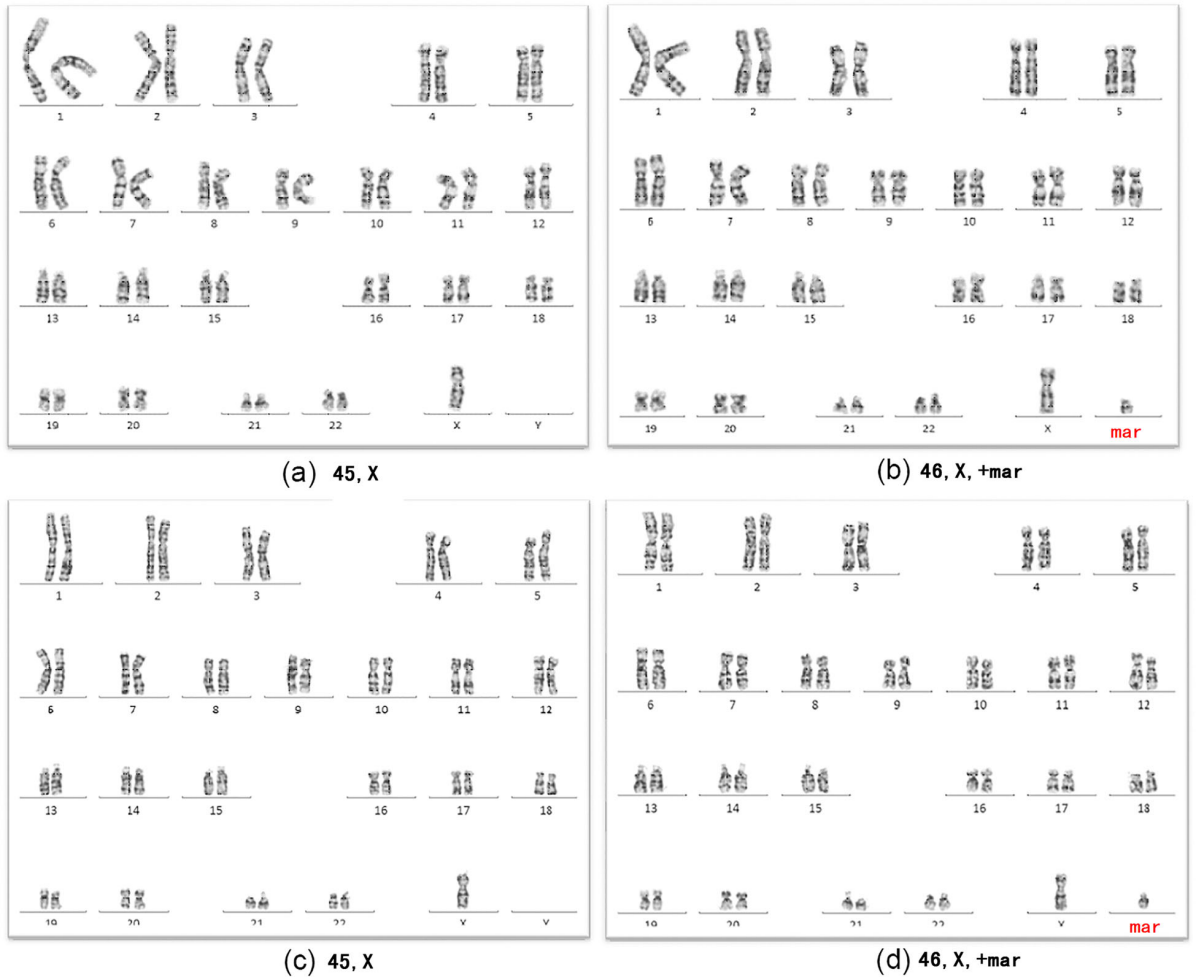
G-banded chromosome karyotyping. Fetus 1: 1a:45,X; 1b:46,X,+mar (**a** and **b**). Fetus 2: 1c:45,X; 1d:46,X,+mar (**c** and **d**). Both karyotypes are chimeras

The fetuses with sSMC (Table 1) were investigated using cytogenetic and molecular cytogenetic means. The chromosome karyotypes of fetuses 1 and 2 were respectively, mos 45,X[92]/46,X,+mar[8] (Fig. 1a and b), and mos 45,X[20]/46,X,+mar[80] (Fig. 1c and d).

**Table 1:** Results of two fetuses with sSMC

Fetus No.	Fetus 1	Fetus 2
Karyotyping	mos 45, X[92]/46, X,+ idic(Y)[8]	mos 45,X[20]/46,X,+ idic(Y)[80]
SNP Array	7.76 Mb deletion in Yq11.222q11.23; 15.68 Mb duplication in Yp11.2q11.21	21 Mb repetition in Yp11.3q11.223
FISH	mos nuc ish(DXZl×1,DYZ3 × 0)[178]/(DXZl×1, DYZ3×2)[22]	mos nuc ish(DXZl×1,DYZ3×0)[38]/ (DXZl×1, DYZ3×2)[162]
AZF	AZFa(+) AZFb+c(−)	AZFa+b + c(+)
Parental comparison	(−)	(−)
Outcome	Pregnancy terminated by no fetal examination.	Normal male born with normal development at 10 months of age

**Fig. 2:**
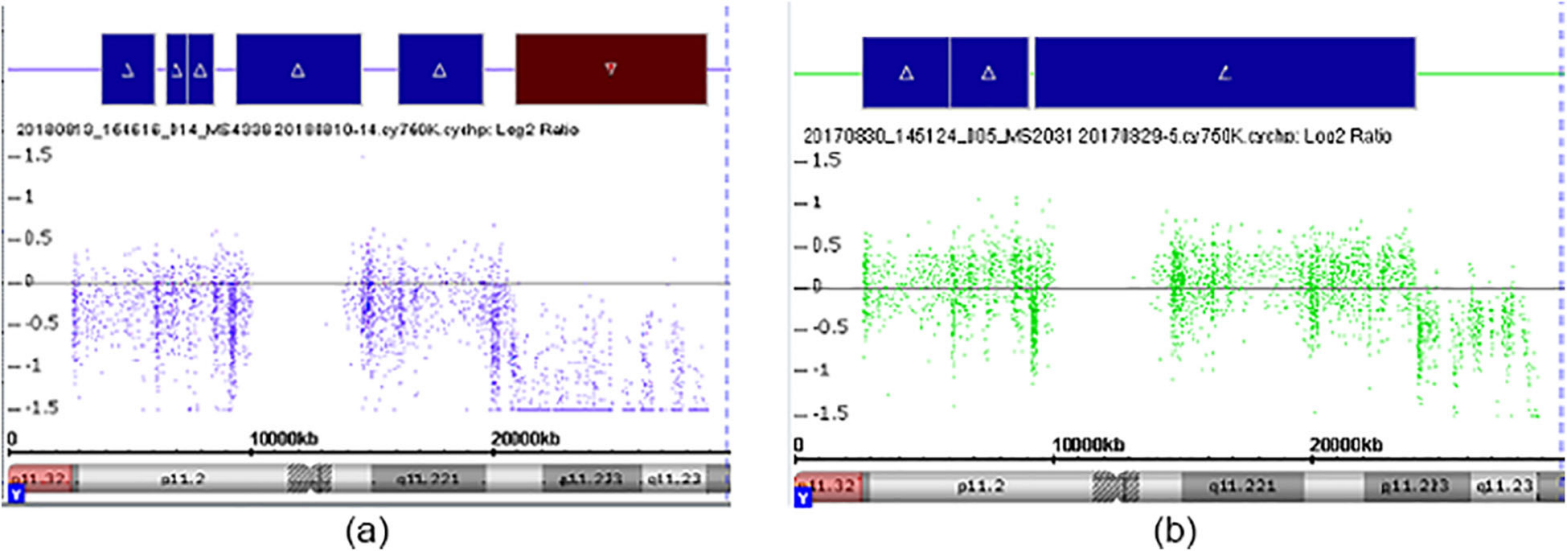
Single nucleotide polymorphism array findings. Red part of fetus 1 (**a**) indicates 7.76 Mb deletion in Yq11.222q11.23; blue part shows 15.68 Mb repeat in Yp11.2q11.21. Blue part of fetus 2 (**b**) shows ~ 21 Mb repeat in Yp11.3q11.223

The SNP array detected a deletion of 7.76 Mb in Yq11.222q11.23 of Case 1, In addition, fetus 1 had a duplication of 15.68 Mb in Yp11.2q11.21. However, reports describing pathogenicity were not found in the ClinGen CNV or DGV databases. A few reports describing pathogenicity with uncertain significance were found in DECIPHER. Fetus 1 had further paternally inherited, autosomal mixed deletions spanning 0.57 Mb at 6q16.2 (Fig. 2a). Approximately 21 Mb were duplicated in Yp11.3q11.223 of fetus 2. However, reports of pathogenicity were not found in ClinGen CNV, DGV or DECIPHER (Fig. 2b).

**Fig. 3:**
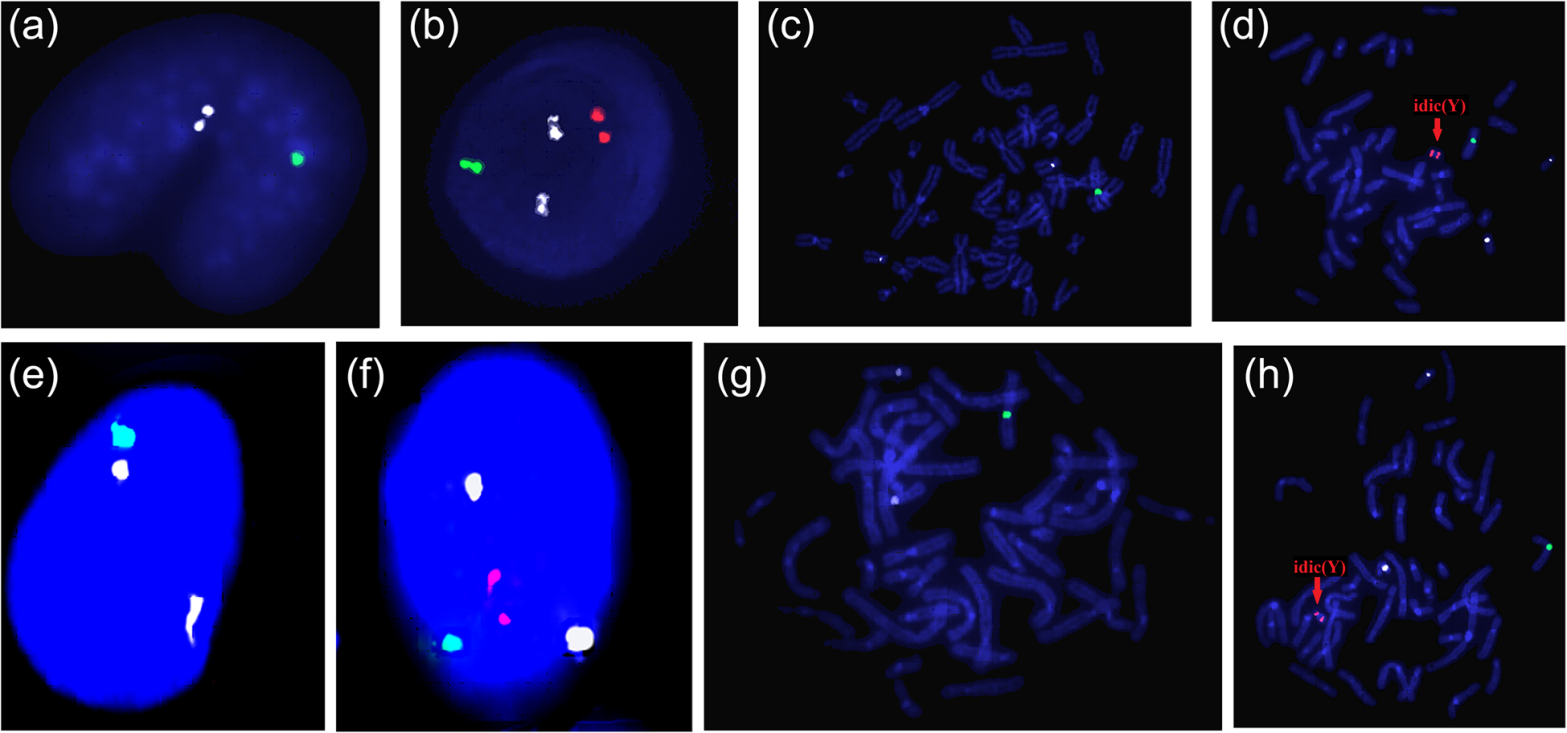
Fluorescence in situ hybridization findings. CEP18 (white) /X (green) /Y (orange) with white signal chromosome 18 as reference. Fetus 1: One X centromeric signal in interphase (**a**); one X centromeric signal and two Y centromeric signals in interphase (**b**); one X chromosome in metaphase (**c**); one X chromosome and one dicentric Y chromosome in metaphase (**d**). Fetus 2. One X centromeric signal in interphase (**e**); one X centromeric signal and two Y centromeric signals in interphase (**f**); one X chromosome in metaphase (**g**); one X chromosome and one dicentric Y chromosome in metaphase (**h**). Both fetal karyotypes are chimeras

The chimeric ratio determined by FISH was consistent with that the karyotypes. In the interphase cells of both fetuses, all green DXZl probes for the pericentromeric region of the X chromosome displayed one hybridization signal, whereas the orange DYZ3 probes for the pericentromeric region of the Y chromosome displayed either two or no hybridization signals. The results of metaphase cells were the same for both fetuses. The two hybridization signals originating from the orange DYZ3 probes were derived from the same small chromosome. The results of fetuses 1 and 2 were respectively, mos nuc ish(DXZl×1,DYZ3×0)[178]/(DXZl×1, DYZ3×2)[22] and mos nuc ish(DXZl×1, DYZ3×0)[38]/ (DXZl×1, DYZ3×2)[162] (Fig. 3).

#### Detection of Y chromosome microdeletions

**Fig. 4:**
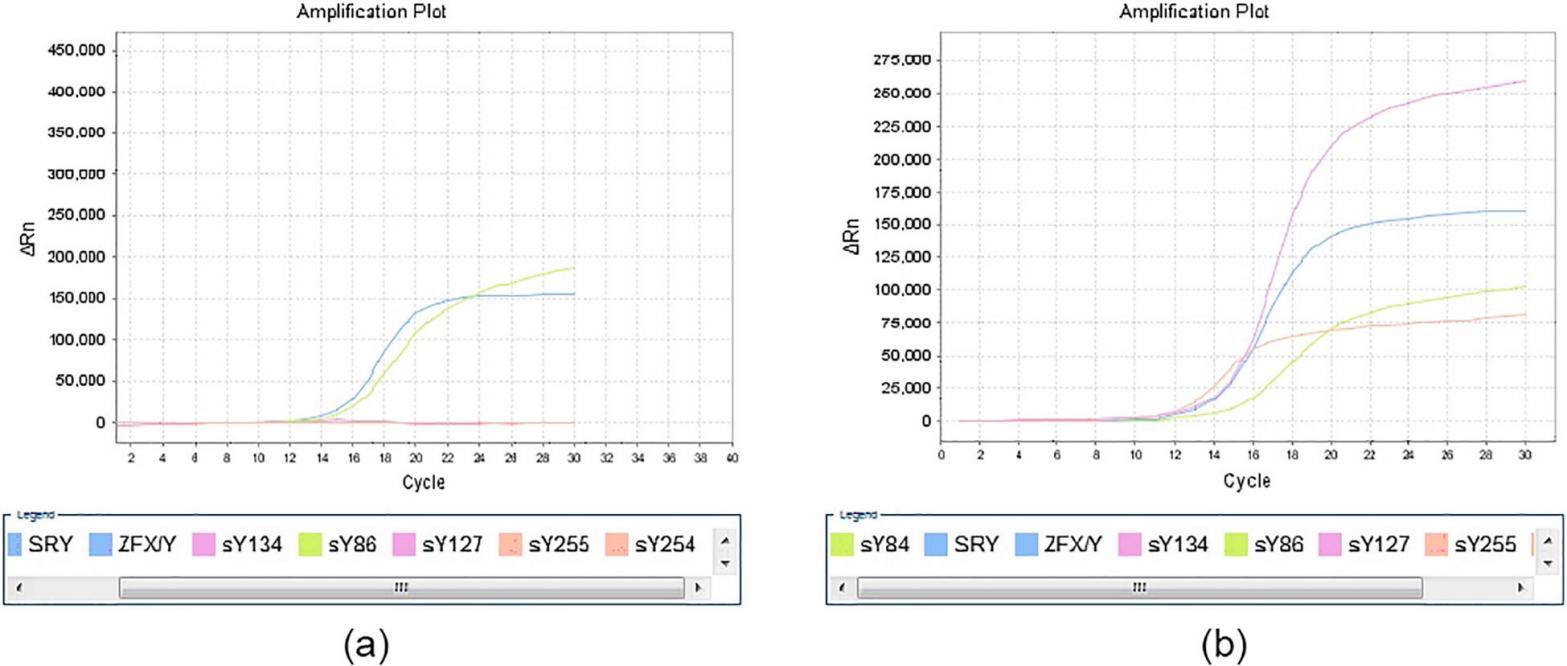
Detection of Y chromosome microdeletions. S-shaped amplification curve of loci of internal references ZFX/Y, sY84 and sY86 in fetus 1 show AZFa region and missing AZFb+c region (**a**). S-shaped amplification curves of all loci in fetus 2, which indicates that AZF showed no deletion (**b**).

Fetus 1 contained the SRY and AZFa regions but lacked the AZFb+c region (Fig. 4a), whereas fetus 2 had all functional regions of SRY and AZF (Fig. 4b).

### Ultrasound and comparisons with parental chromosomes

Ultrasound imaging revealed that both fetuses possessed male external genitalia. The parental chromosomes did not have any abnormalities associated with idic (Y).

### Comprehensive analysis

The above results indicated that both mar carried by the two fetuses were de novo idic (Y). The chromosome karyotypes of fetuses 1 and 2 were respectively, mos 45,X[92]/46,X,+idic(Y)(q11.21)[8] and mos 45,X[20]/46,X,+ idic(Y) (q11.223)[80].

## Discussion and conclusions

Chromosome karyotyping identified sSMC in two fetuses. The incidences of sSMC identified prenatally, among live births and among patients with developmental disorders are 0.4–1.5%, 0.14–0.72%, and 0.288%, respectively. Different origins of sSMC can lead to dicentric, circular, and small segment chromosomes with one centromere [6]. Most sSMC are derived from a gonosome, and some can be derived from an autosomes [7]. Small SMC derived from autosomes mainly form circular chromosomes, followed by small-segment, and a few dicentric chromosomes. Liehr et al. found that 72.6, 27, and 0.4% of sSMC are derived from Y chromosomes, X chromosomes, and autosomes, respectively [8]. The sSMC derived from Y chromosomes respectively present frequently and occasionally as small segment and circular chromosomes. The underlying mechanism through which these sSMC are formed remains unclear.

Since sSMC have no characteristic bands, and traditional chromosome banding techniques cannot recognize its source or characteristics, a combination of multiple detection technologies is needed for analysis [9, 10]. The SNP array revealed a 7.76 Mb deletion in Yq11.222q11.23 and a 15.68 Mb duplication in Yp11.2q11.21 of fetus 1 and approximately 21 Mb of repetitive segments in Yp11.3q11.223 of fetus 2. These data indicated that the sSMC originated from Y chromosomes in both fetuses. Therefore, the repetitive segments might be idic (Y). The FISH results showed that the sSMC of both fetuses had two Y centromeric signals, which further suggested that the sSMC forms were idic (Y). However, the mechanism of idic (Y) formation remains unclear. The popular belief is that when the sister chromatid of Y is separated during mitosis, cleavage occurs in regions of palindromes or inverted repeats. After the homologous exchange of sister chromatids, the cleavage site fuses to form idic (Y). Chromosomal segments without centromeres are degraded [11]. The karyotypes of both fetuses were chimeras that might have developed due to the instability of idic (Y), which is often lost in mitosis, and the subsequent formation of 45,X cells by 46,X, idic (Y) cells. Most Y chromosome abnormalities are inherited paternally, and comparisons of paternal and fetal chromosomes showed that both fetuses had de novo idic (Y).

Lange et al. proposed that the SRY gene is often deleted when a break occurs in the short arm of the Y chromosome, and the phenotypes will be mostly female. When a patient presents as female, they might also have signs of Turner syndrome and streak gonads. In contrast, when a patient presents as they might also have symptoms of azoospermia or oligospermia, asthenospermia, hypospadias and cryptorchidism. Some patients also have gender ambiguity and gonadal dysgenesis [12] and others might also be at higher risk for mental retardation and disorders [11, 13]. Therefore, gender should be determined by ultrasound when prenatal test results show sex chromosome abnormalities in a fetus. Both fetuses described herein had male external genitalia.

The AZF gene located at the distal end of the long arm of Y chromosome was discovered during the 1970s and was later found to be linked to primary azoospermia, severe oligospermia and asthenospermia in males. A microdeletion of AZF is the second leading cause of male infertility, after Klinefelter syndrome [14]. The AZF gene comprises AZFa, AZFb, and the AZFc subregions, and a deletion of one or more of them can cause dyszoospermia. We searched for Y chromosome deletions by probing six STS within the three regions of the AZF gene [15] and found a deleted AZFb+c region and no AZF deletion in fetuses 1 and 2, respectively. Repping et al. proposed that the main cause of a deleted AZFb+c region is that the distal P5 and the proximal P4 of the AZFb region share sequence similarity with the P1 of the AZFc region. When the two ends are broken, homologous recombination occurs, then the AZFb+c region in the center is lost [16]. A deletion of only the AZFc region has mild effects. This region includes the DAZ multigene family that is deleted in azoospermia. Although azoospermia persists, spermatogenesis can still proceed, and sperm can be detected in the testis. Intracytoplasmic sperm injection (ICSI) allows such patients to have offspring. However, the ability for spermatogenesis is lost when a deletion occurs in AZFb, AZFa, or AZFb+AZFa regions, and sperm cannot be recovered from the testis [17, 18].

The above findings indicated that the karyotype of fetus 1 was mos 45,X[92]/46,X,+idic(Y)(q11.21)[8], with the 45,X cell type being the most prevalent. B-ultrasound detected male external genitalia.

After delivery, fetus 1 could present with symptoms of male Turner syndrome, such as short stature, low intelligence, lack of puberty, primary hypogonadism hypoplasia, and gonadal cytoma(http://ssmc-tl.com/ssmc-y-male.html#si) [19–21]. Furthermore, because the AZFb+c region was deleted in this fetus, he would become completely infertile upon reaching adulthood. The fetal karyotype of fetus 2 was mos 45,X[20]/46,X,+idic(Y)(q11.223)[80], with about 21 Mb of repetitive segments in Yp11.3q11.223 of his Y chromosome, which contained the AZF region. Male external genitalia were detected using B-ultrasound. After delivery, this fetus could display the phenotype of XYY syndrome, which is generally normal, except for a possible tendency towards violence or low intelligence. Typical symptoms are not evident during early childhood, and fertility is preserved in adulthood [22]. After genetic counseling provided by Hunan Provincial Maternal and Child Health Care Hospital, the pregnancy of patient 1 was terminated, whereas the fetus of patient 2 was carried to term, delivered, and he has reached the age of 10 months, with normal appearance and growth.

In conclusion, multiple detection strategies should be applied to prenatally distinguish fetuses with congenital malformations or genetic diseases. Moreover, accurate genetic counseling should be provided to reduce the rate of birth defects and improve the health of the population, which is indispensable for prepotency.

## Data Availability

All data generated or analyzed are included in this article.

## Abbreviations

AZF: Azoospermia factor
CNV: Copy number variation
DAZ: Deleted in azoospermia
DECIPHER: Database of Chromosomal Imbalance and Phenotype in Humans using Ensembl Resources
DGV: Database of Genomic Variants
FISH: Fluorescence in situ hybridization
ICSI: Intracytoplasmic sperm injection
ISCN: International System for Human Cytogenomic Nomenclature
OMIM: Online Mendelian Inheritance in Man
SNP: Single nucleotide polymorphism
sSMC: Small supernumerary marker chromosome
STS: Sequence-tagged sites
UCSC: University of California Santa Cruz
